# Dietary Calcium but Not Elemental Calcium from Supplements Is Associated with Body Composition and Obesity in Chinese Women

**DOI:** 10.1371/journal.pone.0027703

**Published:** 2011-12-07

**Authors:** Lina Huang, Jingyi Xue, Ying He, Jian Wang, Changhao Sun, Rennan Feng, Jianhua Teng, Yonghan He, Ying Li

**Affiliations:** 1 Department of Nutrition and Food Hygiene, School of Public Health, Harbin Medical University, Harbin, People's Republic of China; 2 Department of Cardiology, First Affiliated Hospital of Harbin Medical University, Harbin, People's Republic of China; 3 Center for Disease Control and Prevention of Harbin, Harbin, People's Republic of China; Yale University School of Medicine, United States of America

## Abstract

**Objective:**

We assessed whether dietary calcium intake or calcium supplements associated with body composition and obesity in a Chinese population.

**Methods:**

A cross-sectional survey was performed in a population of 8940, aged 20 to 74 y. 8127 participants responded (90.9%). Height, weight, fat mass (FM), waist circumference (WC) and hip circumference were measured. Obesity definition: body mass index (BMI) ≥28 kg/m^2^ (overall obesity); WC ≥85 cm for men or ≥80 cm for women (abdominal obesity І) and waist hip ratio (WHR) ≥0.90 for men or ≥0.85 for women (abdominal obesity П). The data on dietary calcium and calcium supplements were collected using food-frequency questionnaire and self-report questionnaire. Multivariate linear and multivariable logistic regressions were used to examine the associations between dietary calcium intake or calcium supplements and body composition and obesity.

**Principal Findings:**

The average dietary calcium intake of all subjects was 430 mg/d. After adjusting for potential confounding factors, among women only, negative associations were observed between habitual dietary calcium intake and four measures of body composition (*β*, −0.086, *P*<0.001 for BMI; *β*, −0.072, *P*<0.001 for WC; *β*, −0.044, *P*<0.05 for WHR; and *β*, −0.058, *P*<0.01 for FM, respectively) and both measures of abdominal obesity (Odds Ratio [OR] = 0.86, 95% Confidence Interval [CI], 0.80–0.93; *P*<0.001, for abdominal obesity I; OR = 0.92, 95% CI, 0.86–0.99; *P* = 0.026, for abdominal obesity II). These associations were not observed among men (*P*>0.05). Similarly, among both men and women, we did not observe significant associations between calcium supplements and any measures of body composition or abdominal obesity (*P*>0.05).

**Conclusions:**

Dietary calcium from food rather than elemental calcium from calcium supplements has beneficial effects on the maintenance of body composition and preventing abdominal obesity in Chinese women.

## Introduction

Obesity has become a national and global epidemic public health issue during the past decades and it imposes a large societal burden due to increased medical costs. The high prevalence of obesity in the world has stimulated great interest in identifying approaches that may help to prevent weight gain or improve body composition. Increasing calcium intake is one potential means of weight management that has received much attention. As early as 1984, McCarron *et al.* [1] first reported a negative correlation between dietary calcium intake and body weight using cross-sectional data from a National Health and Nutrition Examination Survey. Since then, this report has been supported by many observational studies [2]–[8] and some studies found a gender-specific effect of dietary calcium intake on body composition [7]. Therefore, increased calcium intake by calcium supplementation might be expected to prevent weight gain or improve body composition. However, results from intervention studies have been inconsistent. Few controlled trials have found a significant decrease in body weight in calcium treated subjects [9]–[11], whereas others have not supported this finding in both adults and children [12]–[19].

As is known, the human body has a very strong ability to regulate the absorption and metabolism of calcium. The role of calcium supplements in maintaining body weight remains contradictory. First, intestinal calcium absorption is influenced by calcium intake and the absorption rate of calcium decreases significantly with rising calcium intake. Secondly, subjects had extensive levels of calcium intake at baseline in various trials, especially in certain Western cultures, with more than 1000 mg/d habitual calcium intake in interference studies [15], [20]. We speculate that calcium supplementation plays a small role in body weight in the population due to the higher intake of calcium at baseline. It would be reasonable to validate the effect of calcium supplements on body weight in populations with low calcium intake.

The Chinese have a low level of dietary calcium intake because of diet habits. The data from the National Nutrition Survey of 1992 and 2002 showed that the average dietary calcium intake was only about 405 and 388.8 mg/d, respectively, in the Chinese, which were significantly lower than the Chinese dietary reference intake of calcium (800 mg/d). In recent decades, and especially in the past 10 years, various kinds of calcium products have become commercially available. It is very common to use calcium as a self-administered supplement, especially among Chinese women who are trying to prevent osteoporosis and improve their health conditions. However, there are few studies on the effect of calcium supplements on body composition and obesity prevalence in the Chinese population. The aim of this study was to assess the correlations between habitual dietary calcium intake or use of calcium supplements and body composition variables and obesity prevalence in a Chinese population with native low dietary calcium intake.

## Methods

### Study Participants

The Harbin People's Health Study was a population-based study carried out in 2008 with participants recruited using a stratified multistage random cluster sampling design. This cross-sectional study covered 5 administrative regions of the city. Each region was divided into 3 stratas according to their financial situation. The communities were randomly selected from each stratum in each administrative region, and a total of 15 communities were selected. One to two neighborhood committees were chosen from each of the selected communities. A total of 8940 people, aged 20 y to 74 y, were recruited for this study. In all, 8127 participants responded (90.9%). People were eligible to participate in the study if they: 1) provided written informed consent, 2) were without a history of using postmenopausal hormone therapy, malignancy, thyroid dysfunction, renal calculi, corticosteroid or calcitriol use. Finally, 6712 subjects, consisting of 2433 men and 4279 women, were included in this study. The study protocol was approved by the Ethics Committee of the Harbin Medical University. Each participant signed was informed about the study and signed a consent form. All participants completed a questionnaire-based interview and received a physical examination. Investigators were trained in administering the questionnaire and physical examination before the survey.

### Survey Methods

Demographic data were collected using a standardized questionnaire. Information included age, gender, nation, education, occupation, cigarette smoking (never, past and current), alcohol use, previous medical history and family history. Information on physical activity was collected by using a 1-year physical activity questionnaire [21]. The physical activity level (PAL) was calculated with the formula from the American Institute of Medicine [22]. Dietary data were collected from validated food-frequency questionnaires (FFQs), which contained 103 items, including food and alcohol intake. The energy intake and dietary calcium intake were estimated by the Food Nutrition Calculator (V1.60, Chinese Center for Disease Control [CDC], Beijing, China). Before the survey, a random subsample of 147 healthy subjects completed two FFQs (FFQ1 and FFQ2) and a 3-day dietary record (DR) for assessing the reproducibility and validity of the FFQ. The energy-adjusted correlation coefficient between the two FFQs was 0.75 for dietary calcium and between FFQ2 and DRs was 0.67 for dietary calcium. When dietary calcium intake was categorized by quartile, the proportion of subjects that were classified into the same quartile of calcium intake from the FFQ2 and DR was 43%. Misclassification of subjects into the opposite quartile was 3%. This indicates that the FFQ is a reliable and accurate method for assessing dietary calcium intake. A questionnaire on the use of calcium as a supplement was administered. Respondents self-reported whether they used calcium supplements over the past decade. The information on using calcium supplements included the time, dose, and category of calcium supplements.

The physical examination included measurements of height, fasting body weight, waist and hip circumferences following standard procedures by trained interviewers. Height was measured without wearing shoes by using a steel tape with a maximum of 2 meters and an accuracy of 0.1 meters. Fasting body weight was measured using a portable electronic scale with a dial showing a maximum of 136 kg and an accuracy of 0.5 kg. The electronic scale was placed on a non-carpeted floor, calibrated to zero and participants were without shoes, heavy clothing, and heavy objects before being weighed. Waist circumferences (WCs) and hip circumferences were measured in light clothing, using a tape measure with a maximum of 1.5 meters and an accuracy of 0.1 centimeters. Fat mass (FM) was measured using the electric impedance method with a body FM analyzer (TANITA TBF-300, Tanita Corporation, Tokyo, Japan). The above measurements were performed two times. Body mass indices (BMIs) and waist hip ratios (WHRs) were calculated using the equation: BMI  =  weight (kg)/height (m^2^); WHR  =  waist circumference (cm)/hip circumference (cm) according to the recommendation of the Working Group on Obesity in China (WGOC) [23]. We defined obesity as: 1) BMI ≥28 kg/m^2^ (overall obesity); 2) WC ≥85 cm for men or WC ≥80 cm for women (abdominal obesity І) and WHR ≥0.90 for men or WHR ≥0.85 for women (abdominal obesity П) [23].

### Statistical analysis

Data were presented as mean ± s.d. or percentages as appropriate. Multiple response variables analysis was used to analysis the responses on use of calcium supplements over the past ten years. The associations between dietary calcium intake, use of calcium supplements and continuous outcome variables (BMI, WC, WHR, and FM) were tested by linear regression analysis after adjustment for potential confounders. Multivariable logistic regression models were developed to evaluate the associations between dietary calcium (quartile), use of calcium supplements (yes or no) and obesity after adjustment for age, physical activity level (PAL), energy intake, alcohol use and smoking. The statistical analyses were performed with the Statistical Package for Social Sciences Program (version 13.01S; Beijing Stats Data Mining Co. Ltd). The significance level was set at *P*<0.05 and tests were two tailed for all statistical analyses in this study.

## Results

### Characteristics of subjects

The characteristics of subjects are given in Table 1. There were no significant differences in sunlight time and dietary calcium intake among men and women. The average dietary calcium intake of all subjects was 430 mg/d, and there were merely 1.4% subjects whose daily dietary calcium intake was more than the reference intake of Chinese (800 mg/d). Significant differences were found in other variables (*P<0.001*).

**Table 1:** Characteristics of subjects, values are mean ± SD or n (%).

Variable	Men (2433)	Women (4279)	*P*
Age, yr	48.6±13.0	50.0±12.0	<0.001
Body mass index, kg/m^2^	25.8±3.8	24.8±3.8	<0.001
Waist circumference, cm	87.4±10.1	80.7±10.0	<0.001
Waist hip ratio	0.89±0.07	0.84±0.07	<0.001
Fat mass, kg	17.7±1.7	19.1±1.8	<0.001
Physical activity level	1.43±0.39	1.52±0.41	<0.001
Sunlight time, h/week	10.1±11.8	10.0±10.6	0.763
Energy intake, kcal/d	2371.9±751.0	1955.5±619.1	<0.001
Dietary calcium intake, mg/d	427.6±128.6	431.7±136.5	0.228
Dietary calcium intake >800 mg/d, n (%)	34 (1.4)	58 (1.4)	0.056
Calcium supplementation use, n (%)	536 (22.0)	1643 (38.4)	<0.001
Alcohol use, n (%)	1459 (60.0)	507 (11.8)	<0.001
Smoking, n (%)			<0.001
Never	1040 (42.9)	3980 (93.2)	
Past	280 (11.5)	59 (1.4)	
Current	1106 (45.6)	231 (5.4)	

### The characteristics of participants using calcium supplements over the past decade

There were 536 subjects (22.0%) that had a history of calcium supplement use in men (mean age 48.6±13.0 y, mean BMI 25.8±3.8 kg/m^2^) and 1643 subjects (38.4%) that had a history of calcium supplement use in women (mean age 50.0±12.0 y, mean BMI 24.8±3.8 kg/m^2^) during the past decade.

**Figure 1:**
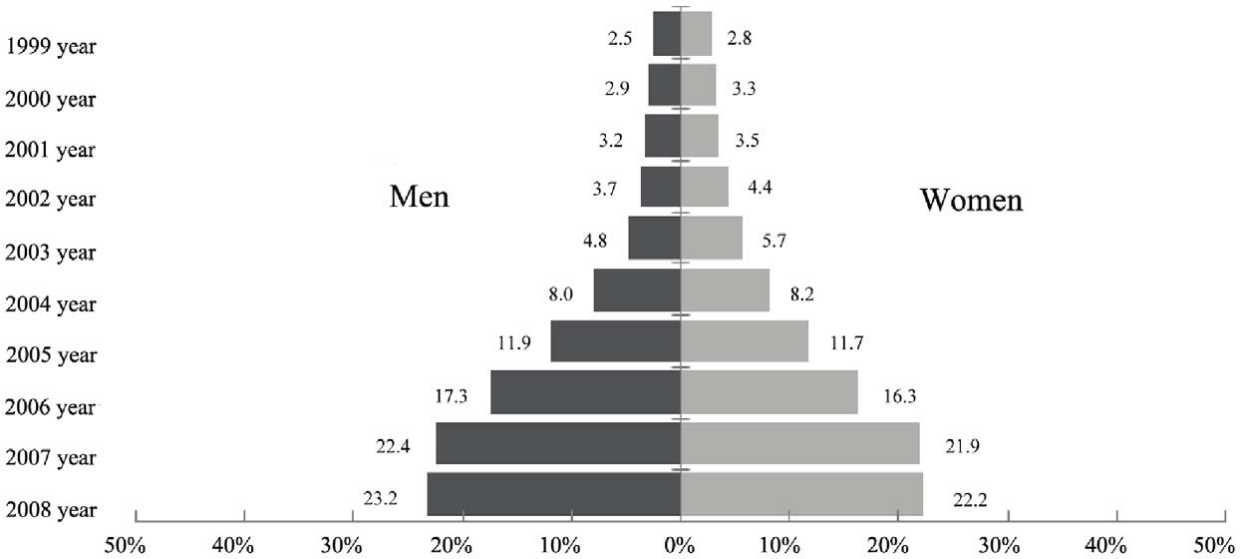
The frequency distribution of responses on calcium supplement use in a Chinese population during 1999–2008.

Figure 1 shows the frequency distribution of responses for calcium supplement use, which was mainly concentrated in the past five years, accounting for 82.8% and 80.3% of the overall responses for the past ten years for men and women, respectively. In the survey, we observed that the dose of calcium supplements was in the range 300 mg/d - 600 mg/d, which was recommended by the calcium product producers, according to the average level of dietary calcium intake of the Chinese.

### Associations between dietary calcium intake and body composition variables and obesity

After adjusting for age, physical activity level, energy intake, smoking, alcohol and calcium supplement use, there was a significant sex-dietary calcium interaction on body composition variables (Table 2, *P<0.05*) and abdominal obesity (Table 3, *P<0.01*). Dietary calcium was found to be inversely associated with BMI (*β*, −0.086, *P<0.001*), WC (*β*, −0.072, *P<0.001*), WHR (*β*, −0.044, *P<0.05*) and FM (*β*, −0.058, *P<0.01*) only in women (Table 2). We also observed that the risk of abdominal obesity was significantly decreased with the increase of each quartile of dietary calcium among Chinese women in multivariable logistic regression models. (Table 3. OR, 0.86; 95% CI, 0.80–0.93; *P<0.001* and OR, 0.92; 95% CI, 0.86–0.99, *P = 0.026*, respectively). However, we did not observe any significant associations between dietary calcium and the same outcome variables among men (Table 2 and Table 3). When considering the data of the subgroup of subjects that did not take calcium supplements, we found that dietary calcium was inversely associated with BMI, WC, WHR and FM. The risk of abdominal obesity was significantly decreased with the increase of each quartile of dietary calcium only in women (data not shown).

**Table 2:** Standardized regression coefficient from the linear regression model.^Δ^

Independent Variables	Dependent Variables			
	BMI (kg/m^2^)	WC (cm)	WHR	FM
Total				
Dietary calcium, mg/d	−0.058**	−0.047**	−0.041*	−0.042*
Calcium supplementation (yes or no)	−0.018	−0.002	−0.003	−0.010
Genger (men = 0, women = 1)	−0.017	−0.231***	−0.240***	−0.274***
Gender*Dietary calcium	−0.081*	−0.092*	−0.083*	−0.087*
Gender* Calcium supplementation	0.041	0.039	0.037	0.030
Men				
Dietary calcium, mg/d	0.009	0.001	−0.030	0.017
Calcium supplementation (yes or no)	−0.023	−0.021	−0017	−0.015
Women				
Dietary calcium, mg/d	−0.086***	−0.072***	−0.044*	−0.058**
Calcium supplementation (yes or no)	−0.017	0.007	0.002	−0.013

*P<0.05.

**P<0.01.

***P<0.001.

Δ Analysis adjusted for age, physical activity level, energy intake, smoking, alcohol use.

**Table 3:** Associations between dietary calcium (quartile) and use of calcium supplements and obesity.

	Overall obesity	Abdominal obesity І	Abdominal obesity П
	Adjusted-OR(95% CI)	Adjusted-OR(95% CI)	Adjusted-OR(95% CI)
Total			
Dietary calcium, quartile	1.01(0.94–1.07)	0.92(0.86–0.97) **	0.95(0.89–1.01)
Calcium supplementation (yes or no)	0.96(0.85–1.10)	0.95(0.85–1.07)	1.01(0.90–1.12)
Genger (0, Men; 1, Women)	0.85(0.72–1.01)	0.70(0.60–0.81) ***	1.07(0.93 –1.23)
Gender* Dietary calcium	0.90(0.80–1.01)	0.88(0.79–0.97) **	0.87(0.79–0.95)**
Gender* Calcium supplementation	1.20(0.91–1.60)	1.26(0.97–1.64)	1.23(0.95–1.61)
Men			
Dietary calcium, quartile	1.09(0.98–1.22)	1.02(0.92–1.13)	1.01(0.92–1.11)
Calcium supplementation (yes or no)	0.92(0.73–1.17)	0.90(0.72–1.12)	0.89(0.73–1.09)
Women			
Dietary calcium, quartile	0.95(0.87–1.04)	0.86(0.80–0.93)***	0.92(0.86–0.99)*
Calcium supplementation (yes or no)	0.96(0.81–1.14)	0.95(0.83–1.09)	1.04(0.91–1.19)

Overall obesity, BMI ≥28 kg/m^2^; Abdominal obesity І, WC ≥85 cm for men or WC ≥80 cm for women; Abdominal obesity П, WHR ≥0.90 for men or WHR ≥0.85 for women.

Multivariate analysis adjusted for age, physical activity level, energy intake, smoking and alcohol use.

*P<0.05.

**P<0.01.

***P<0.001.

### Associations between use of calcium supplements and body composition variables and obesity

**Figure 2:**
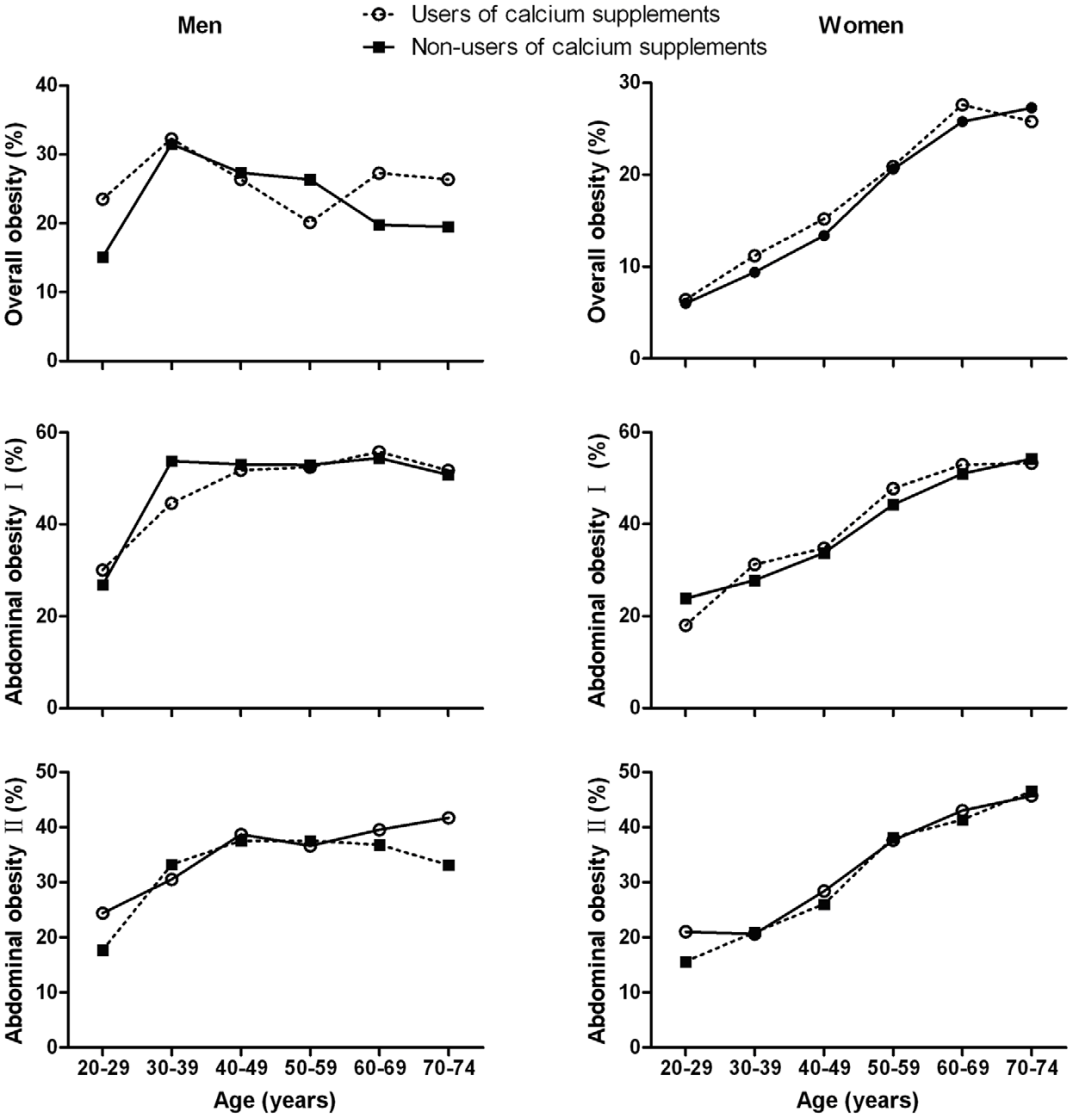
The trend of overall obesity and abdominal obesity in three curves with an increase in age strata. There was no significant difference in obesity rates between users of calcium supplements and non-users of calcium supplements at each age stratum. Overall obesity, BMI ≥28 kg/m^2^; abdominal obesity І, WC ≥85 cm for men or WC ≥80 cm for women; abdominal obesity П, WHR ≥0.90 for men or WHR ≥0.85 for women.

Figure 2 shows that the rates for increasing overall obesity and abdominal obesity had a common trend. There was an increase in age in the two curves for non-users of calcium supplements and users of calcium supplements, respectively. There were no significant differences in obesity rates between users of calcium supplements and non-users of calcium supplements at each age stratum.

After adjusting for potential confounders, we did not find any significant association between use of calcium supplements and body composition variables among the Chinese population in the multivariate linear regression analyses (Table 2). Likewise, we did not observe any impact from the use of calcium supplements on overall obesity and abdominal obesity prevalence among the Chinese population in multivariable logistic regression models (Tables 3 and 4).

**Table 4:** Associations between time of calcium supplement use and obesity after multivariable adjustment.

	Overall obesity	Abdominal obesity І	Abdominal obesity П
	Adjusted-OR (95% CI)	Adjusted-OR(95% CI)	Adjusted-OR(95% CI)
Men			
No use	1.0	1.0	1.0
<12 mo	0.97(0.75–1.26)	0.94(0.74–1.20)	0.90(0.72–1.12)
12-<36 mo	0.79(0.47–1.33)	0.77(0.49–1.22)	0.77(0.50–1.18)
≥36 mo	0.69(0.25–1.90)	0.73(0.31–1.75)	1.42(0.62–3.25)
Women			
No use	1.0	1.0	1.0
<12 mo	0.93(0.77–1.12)	0.93(0.79–1.08)	1.04(0.89–1.20)
12-<36 mo	0.99(0.74–1.30)	0.99(0.78–1.26)	1.07(0.85–1.35)
≥36 mo	1.34(0.87–2.06)	1.09(0.71–1.68)	0.90(0.61–1.32)

Overall obesity, BMI ≥28 kg/m^2^; Abdominal obesity І, WC ≥85 cm for men or WC ≥80 cm for women; Abdominal obesity П, WHR ≥0.90 for men or WHR ≥0.85 for women.

Multivariate analysis adjusted for age, physical activity level, energy intake, smoking, alcohol use, dietary calcium intake.

## Discussion

In this survey, we observed inverse associations between habitual dietary calcium intake and body composition variables in women after adjustment for age, physical activity, energy intake, smoking, alcohol use and calcium supplement use. We also observed that the risk of abdominal obesity was significantly decreased with the increase of each quartile of dietary calcium in multivariable logistic regression models in women after adjustment for the same potential confounders. Previous observational studies have demonstrated inverse relationships between dietary calcium intake and BMI, body fat mass or weight change in Western populations [1]–[8]. In the America National Health and Nutrition Examination Survey NHANES III data set, after controlling for energy intake, an inverse relationship was observed in women (n = 380; P<0.0009) [5].

Different mechanisms were proposed to explain the effect of dietary calcium on body weight changes. Intracellular calcium ([Ca^2+^]_i_) is a key regulator of lipid metabolism. Elevated intracellular calcium concentrations stimulate the expression and activity of lipogenic enzymes and reduce lipolysis with a subsequent increased accumulation of fat in adipocytes [5]. It is well known that lower dietary calcium intake can lead to increased concentrations of 1,25-dihydroxyvitamin D, favoring an increase in [Ca^2+^]_i_ , which promotes lipogenesis. Conversely, a high calcium intake results in lower concentrations of 1,25-dihydroxyvitamin D and an increase in lipolysis [5], [24]. Another possible explanation is that high levels of calcium can reduce absorption of fat in the gut. Some studies in humans and animals have suggested that calcium increases the excretion of fat, presumably by formation of insoluble calcium fatty acid soaps or by binding of bile acids, resulting in malabsorption of fat [25]–[29].

However, we observed that there was a significant sex-dietary calcium interaction on body composition variables and abdominal obesity in a Chinese population. We found the inverse associations between body composition variables, abdominal obesity and dietary calcium intake in Chinese women, but not in men. There have been previous reports of gender-specific effects of calcium intake on body composition. In a study by Jacqmain et al. [7], after adjustment for confounding variables, no relation was observed for men, but a negative relation was observed for women. Loos et al. [30] reported that there were no effects in Black women but a negative relation in Black men, White men and White women. Kamycheva et al. [31] found that there was no effect in men but a positive relation for women. There were no consistent patterns emerging on a possible gender-specific effect of calcium intake on body composition.

In our study, though it is hard to explain why high habitual dietary calcium intake has beneficial effects only in women, a possible explanation may be the effects of women's sex hormones. Abrams [32] and Heaney [33] have recently demonstrated that increases in plasma estrogen concentrations were associated with an increase in intestinal calcium absorption, which may result in significant metabolic changes in women.

Based on the findings from previous studies and the present survey, calcium supplements may have a beneficial effect on body weight. However, many studies indicated that calcium from supplements have few effects on body weight or composition. Shapses *et al.* [15] found that supplementation with 1 g calcium citrate malate or calcium citrate per day during a 25-week weight-loss intervention did not influence body fat or body weight in 100 premenopausal and postmenopausal women. Two systematic reviews [34], [35] of randomized trials of calcium supplementation in adults did not support a beneficial effect in the calcium supplementation group.

In certain intervention studies using calcium supplements, the baseline habitual calcium intake was more than 1000 mg/d, especially in the Western population, which may lead to inconsistent conclusions between surveys and interventional studies. We therefore investigated the effect of the calcium supplements on body weight and fat mass in a Chinese population with low calcium intake. In the present study, we collected data on dietary calcium and calcium supplements using a validated FFQ and self-report questionnaire. The data showed that there was no appreciable association between use of calcium supplements and body composition variables and obesity prevalence in Chinese people after adjusting for age, dietary calcium intake, physical activity level, energy intake, smoking and alcohol use, in spite of the average dietary intake of calcium being only 430 mg/d in the 8940 survey subjects. In addition, the lack of a calcium supplement effect on body weight or fat mass suggests that elemental calcium from supplemental products do not have the expected effects in a Western population with high dietary calcium intake or in a Chinese population with low dietary calcium intake.

There are several aspects that may explain why dietary calcium causes more beneficial effects on male weight or fat mass than calcium supplements. First, dietary calcium is ingested as part of a mixed meal with the presence of other nutrients, such as proteins, carbohydrates and lipids, which are absorbed over a period of several hours as the meal is digested and passes through the small bowel, resulting in a relatively long-term effect on serum calcium. However, people usually take elemental calcium from supplements alone in a fasting state, instead of with meals, which contributes to a transient effect compared to dietary calcium. Secondly, a number of other bioactive compounds in the diet may promote calcium absorption and enhance calcium function. It has been reported that, in some studies, the combination of dairy protein and dietary calcium enhanced weight loss more effectively than either compound alone [5], [36]. Thirdly, as described above, calcium can reduce the absorption of fat in the gut, which is achieved when fat and calcium co-exist in the intestine, which may explain, at least partly, why dietary calcium can promote fat excretion more effectively. Taken together, the source of calcium from foods rather than elemental calcium from supplements had a profound effect on body composition and obesity.

The inferences presented in this cross-sectional study are limited due to the observational and descriptive nature of this evidence. In this survey, we observed that the frequency distribution of responses on calcium supplement use was mainly concentrated in the past five years (about 80%), which may be due to calcium products becoming increasingly available with increasing propaganda and public awareness of healthcare. If there is any effect of calcium supplements on body composition, it should be readily observed because of the recent and concentrated advertisement for calcium supplements. In addition, all information on calcium supplement use was self-reported, thus there was the potential for misclassification of calcium supplement use.

In conclusion, we observed that habitual dietary calcium intake, but not use of calcium supplements, was inversely associated with body composition and abdominal obesity in Chinese women. It is possible that the beneficial effect of calcium on body composition and obesity could be significant if it is taken with naturally calcium-rich content food. Because of the observational nature of the present study, our findings need to be interpreted with caution. Whether dietary calcium, but not elemental calcium from supplements, has beneficial effects on the maintenance of body composition and the prevention of abdominal obesity in Chinese women needs to be assessed in long-term studies.
